# The role of MCT1 in tumor progression and targeted therapy: a comprehensive review

**DOI:** 10.3389/fimmu.2025.1610466

**Published:** 2025-06-19

**Authors:** Zheng Xu, Xuemei Wang, Hongjing Cheng, Jiuling Li, Xin Zhang, Xueju Wang

**Affiliations:** Department of Pathology, China-Japan Union Hospital, Jilin University, Changchun, Jilin, China

**Keywords:** MCT1, oncogenic mechanism, tumor progression, MCT1 inhibitor, targeted therapy

## Abstract

Overexpression of monocarboxylate transporter 1 (MCT1) in tumor cells is often associated with poor prognosis. The established mechanisms through which MCT1 and its mediated lactate transport drive tumor progression are manifold. The classical mechanisms include fostering metabolic symbiosis among tumor cells, dampening the immune function of immune cells, and spurring tumor angiogenesis. Beyond these, new findings of MCT1’s role in tumor progression have emerged. These new findings highlight MCT1’s involvement in mediating the reverse Warburg effect, inhibiting ferroptosis, promoting protective autophagy, and augmenting tumor glycolysis. When acetate serves as a transport substrate for MCT1, additional mechanisms come into play. These encompass MCT1’s participation in the acetylation of histone H3K27 and its role in upregulating c-Myc levels. Several studies have demonstrated that while selective MCT1 inhibitors can effectively impede tumor progression, they also face notable challenges. To address these, combining MCT1 inhibitors with other drugs appears to hold more promise.

## Introduction

Aerobic glycolysis is a hallmark of malignant tumor metabolism (1), where tumor cells prefer this process over oxidative phosphorylation for energy production, even in the presence of ample oxygen (2). This metabolic preference leads to the production and extracellular release of lactate, which acidifies the tumor microenvironment (TME) (3). Members of the monocarboxylate transporter (MCT) family, particularly MCT1, play a crucial role in tumor progression by facilitating lactate transport (4). MCT1, encoded by the SLC16A1 gene (5), is one of the 14 MCTs(MCT1-14) family members and is expressed in nearly all human tissues (6). Its expression and function are primarily regulated by p53 and MYC (7). Research by Boidot et al. demonstrated that p53 directly interacted with the MCT1 gene promoter to inhibit its expression (8). In a study involving neuroblastoma and Burkitt’s lymphoma by Gan et al, MYC directly activated transcription of the MCT1 by binding to a specific recognition site of the gene (9). Additionally, MYC repressed the transcription of miR29a and miR29c, which in turn increased the expression of their target protein, MCT1 (9). CD147, a transmembrane glycoprotein (10), is often co-expressed with MCT1 (11). MCT1 can function as a substrate transporter when it binds to CD147 (4). MCT1 facilitates the transmembrane transport of various substrates, including lactate (12), acetate (13), pyruvate (12), butyrate (14), ketone bodies (15), β-hydroxybutyrate (16), and succinate (17). Although MCT1 can transport multiple substrates, its primary physiological role is to mediate lactate entry into cells and, in conjunction with monocarboxylate transporter 4 (MCT4), lactate export out of cells (1). This process is modulated by the cell’s metabolic state and the intra- and extracellular concentrations of lactate and protons (15). Lactate, while not the only substrate for MCT1, is the most extensively studied and prevalent *in vivo*, particularly in tumors, where concentrations can reach up to 40 mM (4).

MCT1 is often indicative of a poor prognosis in patients with malignant tumors, as its expression in tumor cells is frequently associated with reduced survival rates. Typically, MCT1 is localized to the cell membrane. However, in some studies, MCT1 has also been detected in the cytoplasm or nucleus. For instance, in a study involving soft tissue sarcomas by Pinheiro et al, different localizations of MCT1 were found to predict varying prognoses: cytomembrane localization of MCT1 was linked to a poor prognosis, while nuclear localization was associated with a better outcome (18). Similarly, in a study involving diffuse large B-cell lymphoma by Afonso et al, MCT1 was found in the nucleus in a few samples (19). Due to the limited number of samples in this research, the relationship between nuclear expression of MCT1 in tumor cells and prognosis remained unclear. **Table 1** provides a summary of the relationship between MCT1 expression patterns in tumor cells and the prognosis.

**Table 1:** Association of MCT1 expression patterns with prognosis.

Tumor type	Prognosis-related MCT1 location	MCT1 expression level	Association with prognosis	Sample Size	Reference
Gastrointestinal stromal tumors	cytoplasm	high	poor OS	64	(87)
Bladder carcinoma	membrane	high	poor OS	360	(88)
Urothelial bladder carcinoma	membrane and cytoplasm	high	poor DFS and OS	114	(89)
Prostate cancer	membrane	high	poor clinical outcome	480	(90)
Soft tissue sarcomas Soft tissue sarcomas	membrane nuclear	high high	poor OS good OS	86 86	(18) (18)
Osteosarcoma	membrane	high	poor OS	61	(91)
Clear cell renal cell carcinoma	membrane	high	poor PFS	180	(92)
Clear cell renal cell carcinoma	cytoplasm	high	poor PFS	150	(93)
Clear cell renal cell carcinoma	membrane	high	poor OS and CSS	207	(94)
Adrenocortical carcinomas	membrane	/	poor OS	78	(95)
Cutaneous melanoma	membrane	/	poor OS	356	(96)
Oral cavity cancer	membrane	high	poor OS	136	(97)
Hodgkin lymphoma	membrane	high	poor PFS	22	(98)
Endometrial cancer	membrane and cytoplasm	high	poor OS, RFS and CSS	90	(99)
Breast carcinomas	membrane	high	poor PFS	257	(100)
Esophageal squamous cell carcinoma	membrane	high	poor OS and PFS	103	(101)
Glioblastoma	membrane	high	poor OS	1226	(102)
Head and neck squamous cell carcinoma	membrane	/	poor OS and PFS	82	(103)
T-cell non-Hodgkin lymphoma	membrane and cytoplasm	high	poor OS and PFS	38	(104)
Non-small cell lung cancer	membrane	high	good DSS	335	(105)
Pancreatic ductal adenocarcinoma	membrane	high	good OS and PFS	240	(106)

CSS, cancer-specific survival; DSS, disease-specific survival; OS, overall survival; PFS, progression-free survival; RFS, recurrence-free survival;/, not mentioned.

Although the classical mechanisms of MCT1 involvement in tumor progression have been elucidated, tumor-promoting studies of MCT1 through novel mechanisms, such as ferroptosis, remain scattered. In this paper, we will systematically review the multidimensional role of MCT1 in tumor progression and explore potential strategies for its targeted therapy.

## Classical mechanisms of MCT1 in tumor progression

**Figure 1:**
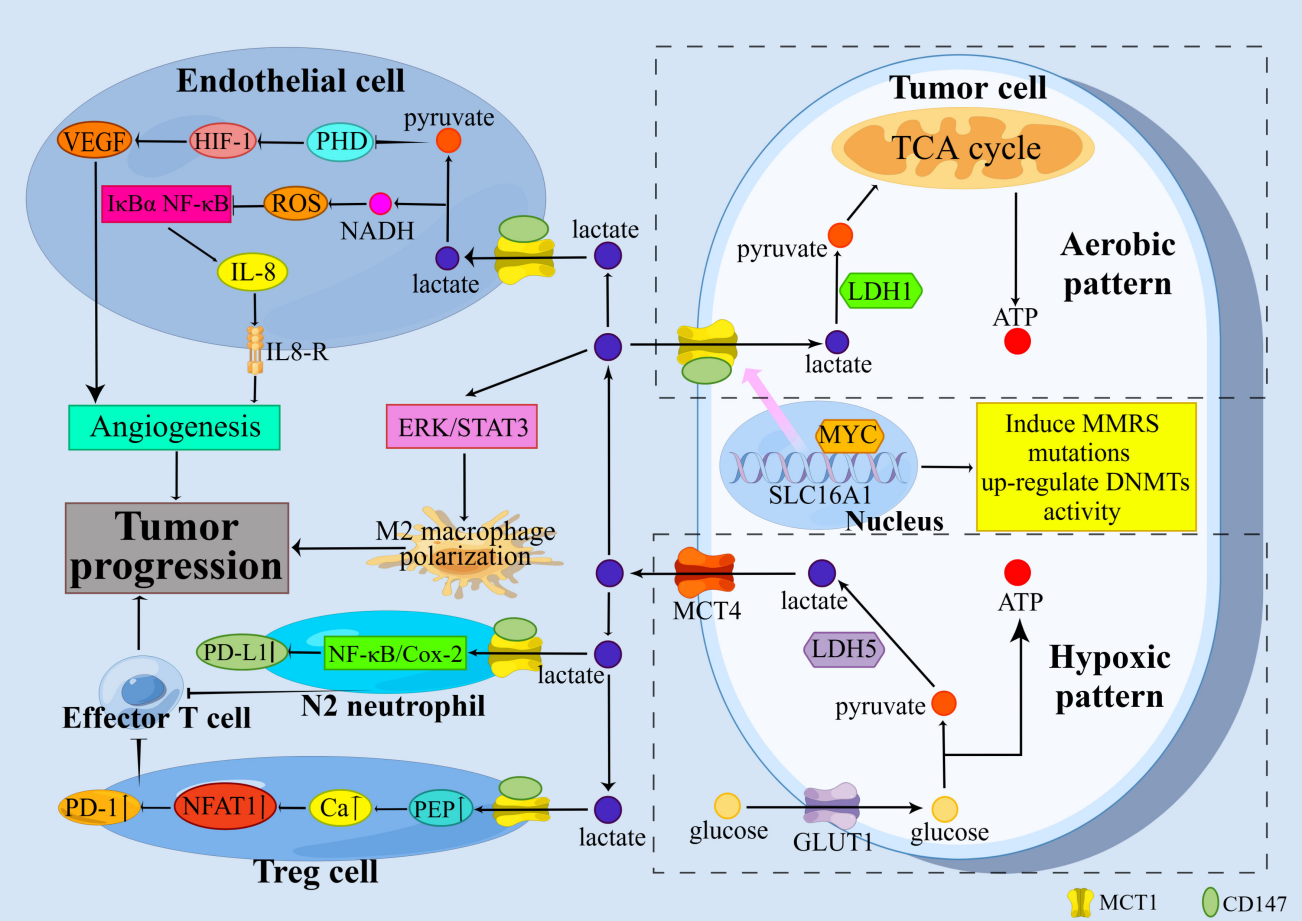
Classical mechanisms by which MCT1 promotes tumor progression. MCT1 promotes tumor progression through the promotion of metabolic symbiosis among tumor cells, the suppression of immune function in immune cells, and the promotion of tumor angiogenesis.

SLC16A1, the gene encoding MCT1, appears to function as a proto-oncogene. SLC16A1 induces mutations in mismatch repair (MMR) genes and increases the activity of DNA methyltransferases (DNMTs) in urologic tumors (5). Elevated levels of DNMT are important for tumor progression (20), as are MMR gene mutations (21). In tumor-related studies, MCT1 and its mediated lactate transport can contribute to tumor progression by promoting metabolic symbiosis among tumor cells, inhibiting the immune function of immune cells, and promoting tumor angiogenesis. **Figure 1** summarizes the classical mechanisms of MCT1 in tumor progression.

### MCT1 mediates metabolic symbiosis

The Warburg effect, characterized by a preference for aerobic glycolysis over oxidative phosphorylation, significantly enhances the aggressiveness of tumor cells exhibiting this metabolic phenotype (3). According to the metabolic symbiosis hypothesis, tumor cells can be divided into two distinct subtypes: oxygenated tumor cells, which are located near blood vessels, and hypoxic tumor cells, which are situated further away from blood vessels (22, 23). To address ATP deficiency and enhance glucose uptake, hypoxic tumor cells upregulate the expression of GLUT1. Hypoxia-inducible factor-1 (HIF-1) facilitates the conversion of glucose to pyruvate and subsequently to lactate in the presence of lactate dehydrogenase-5 (LDH-5). This process promotes the regeneration of nicotinamide adenine dinucleotide (NAD^+^), which is essential for maintaining a high glycolytic flux. Lactate and hypoxia-stimulated hypoxia-inducible factor-1α (HIF-1α) or WNT/β-catenin signaling prevent pyruvate from entering the TCA cycle and converting to acetyl-CoA by upregulating pyruvate dehydrogenase kinase 1 (PDK1) and then inhibiting pyruvate dehydrogenase complex (PDC). This drives tumor cells to gain energy through aerobic glycolysis. Finally, MCT4 removes lactate produced in hypoxic tumor cells to avoid intracellular acidification. Oxygenated tumor cells transport lactate produced by hypoxic tumor cells through MCT1 and produce ATP by oxidizing lactate (24).

Oxidative lactate metabolism offers several advantages to oxygenated tumor cells compared to glucose-dependent respiration. Specifically, ATP production through lactate oxidation is 7.5 times greater than that produced through aerobic glycolysis. Tumor cells prioritize lactate utilization to conserve energy required for the synthesis and maintenance of glycolytic enzymes, as well as for the phosphorylation of glucose and fructose-6-phosphate (F6P) during glycolysis. Additionally, the oxidation of lactate by LDH-1 generates ample nicotinamide adenine dinucleotide (NADH), which serves as a fuel source for the mitochondrial electron transport chain (ETC). Ultimately, the LDH-1 catalyzed reaction may facilitate lysosomal acidification and the maturation of autophagy vesicles. Autophagy allows oxidized proteins and organelles to be recycled (4).

### MCT1 leads to immunosuppression

In addition to its role in metabolic regulation, MCT1 also promotes tumor progression by modulating the immune microenvironment.

Macrophages can differentiate into either M1 or M2 phenotypes (25), M1 macrophages exhibit anti-tumor properties, while M2 macrophages promote tumor growth (26). Tumor cells release lactate via MCT1 or MCT4, which can influence the polarization of M2 macrophages (27). In a study involving breast cancer by Mu et al, it was observed that tumor-derived lactate facilitated the polarization of M2 macrophages through the activation of the extracellular regulated protein kinase (ERK)/signal transducer and activator of transcription 3 (STAT3) signaling pathway (28). In addition, M2 macrophages were found to release immunosuppressive cytokines that can stimulate the development of regulatory T cells (Treg cells) (29).

Treg cells suppress anti-tumor immune responses, while effector T cells play a crucial role in combating tumors (30, 31). The TME facilitates the recruitment and differentiation of Treg cells by upregulating forkhead box proteins P3 (FOXP3) and MCT1. Increased FOXP3 expression enhances the adaptability of Treg cells to the high-lactate TME by suppressing c-Myc and glycolysis, while promoting oxidative phosphorylation (OXPHOS) and increasing NAD^+^ oxidation (32). Treg cells uptake lactate via MCT1, where it is metabolized intracellularly to phosphoenolpyruvate (PEP). This metabolic process leads to an increase in cytoplasmic calcium ion concentration, facilitating the translocation of nuclear factor of activated T-cells 1 (NFAT1) to the nucleus. This enhances the expression of programmed death-1 (PD-1), while PD-1 expression in effector T cells is inhibited (33). PD-1 blockade invigorates PD-1-expressing Treg cells, leading to treatment failure (33). Activated cytotoxic T lymphocytes (CTLs) predominantly utilize glycolysis for energy production, with the resulting lactate being excreted via MCT1 to sustain their cytokine production and cytotoxic functions (34). Elevated lactate levels within the TME impede T cell function by suppressing lactate efflux (35), thereby creating a conducive environment for tumor cell survival.

Neutrophils, similar to macrophages, exhibit two distinct phenotypes: N1 and N2 (36). The N1 phenotype is characterized by cytotoxicity and anti-inflammatory properties, while the N2 phenotype demonstrates immunosuppressive capabilities (36). It has been shown that the transforming growth factor-β (TGF-β)/Smad3 signaling pathway plays a crucial role in the transformation of neutrophils into the N2 phenotype (37). Tumor-derived lactate enters neutrophils via MCT1 and triggers PD-L1 expression through the nuclear factor-kappa B (NF-κB)/cyclooxygenase-2 (COX-2) pathway. Neutrophils expressing PD-L1 have the potential to suppress T cell cytotoxicity (38), thereby facilitating tumor progression.

### MCT1 mediates tumor angiogenesis

In oxygenated tumor cells and endothelial cells, lactate is transported into the cell via MCT1 and subsequently oxidized by LDH-1 to produce pyruvate. Pyruvate is characterized as a pseudo-hypoxic signal that influences HIF prolyl hydroxylases (PHDs), particularly PHD2. When oxygen, α-ketoglutarate, and vitamin C are present, this family of enzymes catalyzes the hydroxylation of HIF-1α on two proline residues, targeting this HIF-1 subunit to proteasome-mediated degradation. This hydroxylation leads to the proteasome-mediated degradation of the HIF-1α subunit. Thus, even in the presence of sufficient oxygen, PHDs can be inhibited by oxidants or α-ketoglutarate competitors. The inhibitory effect of pyruvate on PHDs varies by cell type. In oxygenated tumor cells and endothelial cells, pyruvate stably oxidizes HIF-1α, thereby activating HIF-1 and triggering the transcription of vascular endothelial growth factor A (VEGF-A) in tumor cells and VEGF receptor 2 (VEGFR2) as well as basic fibroblast growth factor (bFGF) in endothelial cells (4). Additionally, endothelial cells possess an autocrine pathway for angiogenesis. Lactate uptake by endothelial cells induces the phosphorylation of the inhibitor of κBα (IκBα), leading to the activation of the NF-κB/interleukin-8 (IL-8) signaling pathway, which promotes angiogenesis in tumor cells (22, 39). VEGF-A, VEGFR2, bFGF, and IL-8 are all pro-angiogenic factors that activate their respective receptors to promote neovascularization (24).

## New findings that MCT1 promotes tumor progression

**Figure 2:**
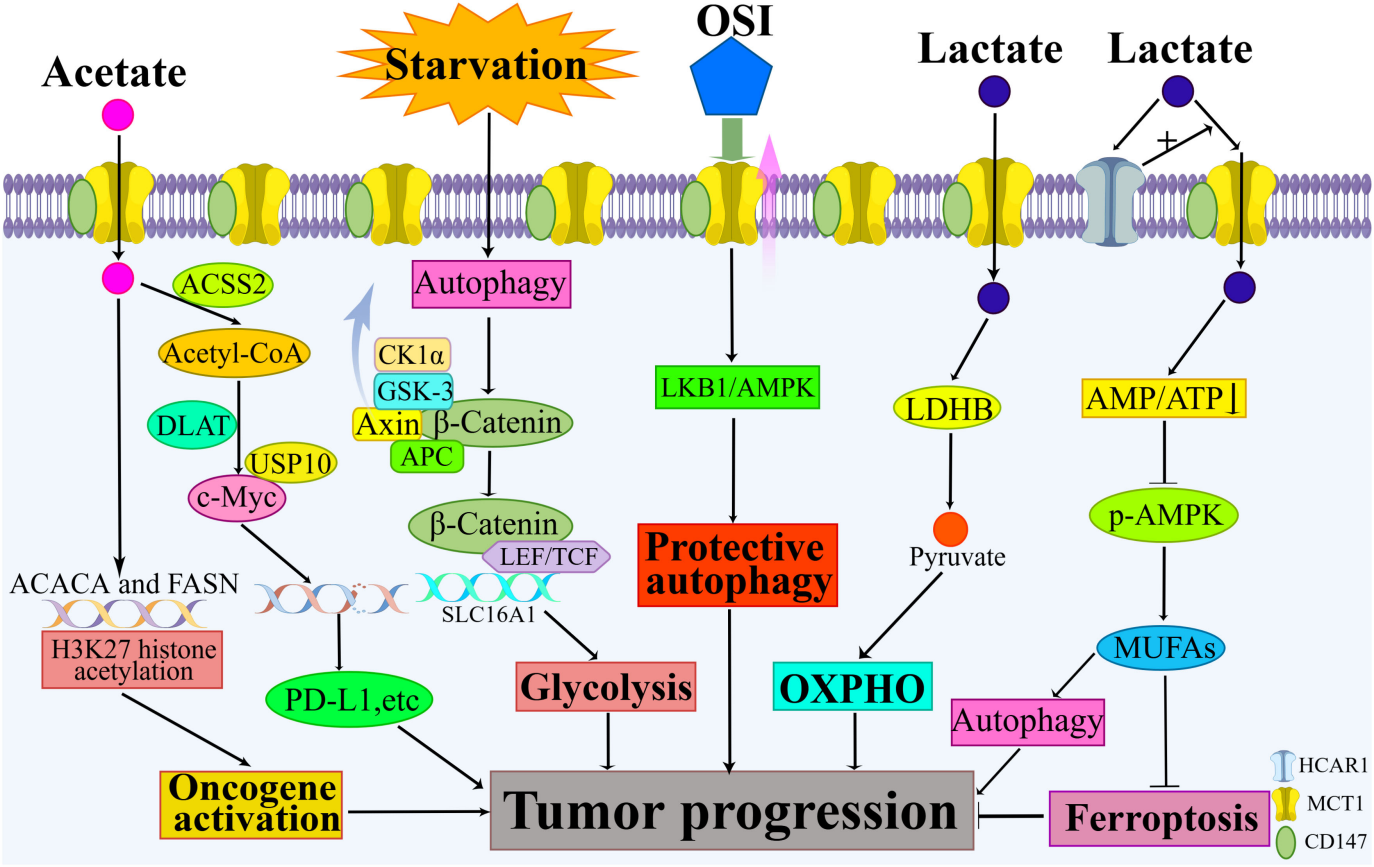
New findings on how MCT1 promotes tumor progression. New findings highlight MCT1’s involvement in mediating the reverse Warburg effect, inhibiting ferroptosis, promoting protective autophagy, and augmenting tumor glycolysis. When acetate serves as a transport substrate for MCT1, additional mechanisms come into play, including MCT1’s participation in the acetylation of histone H3K27 and its role in upregulating c-Myc levels.

Beyond the classical mechanisms of tumor progression that have been previously described, recent studies have unveiled novel roles of MCT1, thereby further elucidating its multifaceted contributions to tumor biology. **Figure 2** summarizes the new findings that MCT1 promotes tumor progression.

### MCT1 mediates reverse Warburg effect

The reverse Warburg effect is observed in the interaction between tumor cells and stromal cells. Hydrogen peroxide released by tumor cells induces oxidative stress in stromal cells. This oxidative stress activates hypoxia-inducible factor-1α (HIF-1α) and nuclear factor-kappa B (NF-κB). HIF-1α not only triggers aerobic glycolysis and angiogenesis but also induces autophagy and lysosomal degradation, leading to the loss of caveolin-1 (Cav-1). The loss of Cav-1 amplifies oxidative stress through a positive feed-forward control mechanism and contributes to metabolic changes in stromal cells. As a result, proteins such as MCT1 are highly activated. Lactate produced by stromal cells enters tumor cells via MCT1 for metabolism (40). This metabolic interaction promotes the creation of a nutrient-rich microenvironment that allows tumor cells to meet their metabolic needs (41).

### MCT1 promotes tumor progression through associated programmed cell death

Obstruction of ferroptosis favors tumor progression. Ferroptosis is a novel form of programmed cell death (42). The primary feature of ferroptosis is the buildup of lipid peroxides (43). Cellular lipids and lipid metabolism play a pivotal role in regulating ferroptosis (44). Hydroxy-carboxylic acid receptor 1 (HCAR1), a member of the G protein-coupled receptor family (45), acts as a receptor for lactate and modulates its metabolism (46). HCAR1 is expressed on the membrane of various cells, including tumor cells, and its activation through lactate binding results in increased MCT1 expression, thereby facilitating lactate uptake by tumor cells (45). In a study involving hepatocellular carcinoma cells by Zhao et al, these cells exhibited resistance to ferroptosis induced by common ferroptosis inducers. The lactate taken up by MCT1 potentially led to increased ATP production and a decreased AMP: ATP ratio within cellular compartments (47). Disruption of the AMP: ATP balance by lactate can deactivate adenosine monophosphate-activated protein kinase (AMPK), upregulate the expression of sterol regulatory element-binding protein 1 (SREBP1) and its target stearoyl-CoA desaturase 1 (SCD1), ultimately resulting in increased synthesis of anti-ferroptosis monounsaturated fatty acids (MUFAs) and reduced lipid peroxidation (47). Furthermore, MUFAs have been shown to enhance the fluidity and curvature of the lipid bilayer, promote the formation of autophagosomes on the endoplasmic reticulum, and activate autophagy (48). Autophagy can counteract cellular lipotoxicity to some extent and is particularly important for the survival of tumor cells (48).

Evidence suggests that autophagy is indispensable for the progression of malignant tumors in numerous instances (49). Osimertinib (OSI), an epidermal growth factor receptor tyrosine kinase inhibitor (EGFR-TKI) (50), has been found to upregulate MCT1 expression in colorectal cancer (CRC) cells (51). This upregulation activated the liver kinase B1 (LKB1)/AMPK signaling pathway, inducing protective autophagy in CRC cells (51). In a study involving hepatocellular carcinoma by Colozza et al, researchers discovered that starvation induced autolysosome production, which activated the Wnt/β-catenin signaling pathway and subsequently upregulated MCT1 expression. This enhanced glycolysis and facilitated tumor metastasis (52). Mechanistically, β-catenin served as a crucial second messenger in the classical Wnt signaling pathway. Under normal physiological conditions, the destruction complex—comprising Axin, adenomatous polyposis coli (APC), glycogen synthase kinase 3 (GSK3), and casein kinase 1α (CK1α)—actively facilitated β-catenin turnover via a proteasome-dependent mechanism. GSK3β-mediated phosphorylation led to the eventual degradation of β-catenin (53). Activation of Wnt signaling led to the binding of the Wnt ligand to its cognate receptors, frizzled (FZD) and lipoprotein receptor-related protein 5 and 6 (LRP5/6). This binding initiated the assembly of a multiprotein complex known as the signalosome and suppressed the activity of the destruction complex (53). The subsequent internalization of the signalosome into early endosomes (EEs), which then matured into multivesicular bodies (MVBs), was essential for the propagation of Wnt signals (53). Consequently, β-catenin was stabilized and translocated to the nucleus, where it cooperated with T-cell factor/lymphoid enhancer-binding factor (TCF/LEF) to activate the transcription and translation of the MCT1 gene (53).

### MCT1-mediated acetate transport promotes tumor progression

When used as a substrate for MCT1, acetate can promote tumor progression through various mechanisms. In a study involving clear cell renal cell carcinoma (ccRCC) by Li et al, researchers identified MCT1 as a significant facilitator of ccRCC, with its role in metabolic reprogramming mediated through acetate transport (54). The transportation of acetate into the cell via MCT1, followed by its translocation into the nucleus, resulted in the activation of oncogenes by enhancing the acetylation of histone H3K27 in the promoter region of the FASN and ACACA genes, ultimately promoting tumor progression (54). In a study involving non-small cell lung cancer by Wang et al, the uptake of acetate by tumor cells, facilitated by high expression of MCT1 and catalyzed by ACSS2 to produce acetyl-CoA, increased lipid synthesis in the tumor cells and led to elevated levels of lysine acetylation at position 148 of c-Myc (13). Most importantly, the researchers found that dihydrolipoamide S-acetyltransferase (DLAT) within the pyruvate dehydrogenase complex (PDC) performed a nonclassical metabolic function and mediated the acetylation of c-Myc (13). Acetylated c-Myc increased binding to the deubiquitinating enzyme USP10, promoting c-Myc protein stabilization and subsequent transcriptional activation of PD-L1, LDHA, MCT1, and Cyclin D1 expression (13). Both the mouse *in situ* tumorigenic lung cancer model and the subcutaneous tumorigenic model demonstrated that the consumption of acetate-containing drinking water inhibits CD8+ T cell infiltration and promotes tumor growth (13).

## Advances in MCT1-related inhibitors

### Inhibition of MCT1 impedes tumor progression

Given its pivotal role in tumor progression, MCT1 has emerged as a promising therapeutic target. Various research groups have explored strategies to inhibit MCT1, employing both genetic knockdown techniques and pharmacological inhibitors.

Inhibiting MCT1 disrupts the metabolic symbiosis between tumor cells. When MCT1 is inhibited using α-cyano-4-hydroxycinnamate (CHC) or siRNA, lactate transport in oxygenated tumor cells ceases. As a result, these oxygenated tumor cells must increase glucose consumption to compensate for the lack of lactate. This disruption in metabolic symbiosis between oxygenated and hypoxic tumor cells leads to increased glucose uptake by oxygenated tumor cells from nearby blood vessels. Consequently, hypoxic tumor cells experience glucose deprivation, leading to apoptosis. The inhibition of MCT1 in oxygenated tumor cells indirectly causes the death of hypoxic tumor cells (55). This underscores MCT1 as a promising target for tumor therapy, as its inhibition not only eliminates hypoxic tumor cells but also allows for the treatment of more vulnerable oxygenated tumor cells through methods like chemotherapy or radiation (23).

Inhibition of MCT1 can suppress tumor angiogenesis. Since MCT1 is a key transporter for lactate uptake by endothelial cells, a study has employed CHC and siRNA to inhibit MCT1, thereby effectively suppressing lactate-induced tumor angiogenesis. Mechanistically, targeted inhibition of MCT1 in endothelial cells directly impeded angiogenesis by reducing HIF-1 activity (56).

Inhibition of MCT1 holds potential to impede tumor progression by reducing immunosuppression. Lactate, a hallmark metabolite of TME (57), actively promotes immune evasion of tumor cells by inhibiting immune cell toxicity and proliferation (58). Tumor-derived lactate has been identified as an inhibitor of CD8+ T cell toxicity (59). A nanomedicine containing MCT1 inhibitors released the drug at low pH, inhibiting MCT1 to curb lactate release and thereby enhancing the anti-tumor activity of CD8+ T cells (60).

Moreover, inhibiting MCT1 prevents tumor cells from absorbing lactate from stroma cells, thereby diminishing the role of stroma cells in promoting tumor cell proliferation. Additionally, inhibiting MCT1 using siRNA or MCT1 inhibitors promotes ferroptosis, an effect that is primarily mediated through the regulation of intracellular lipid metabolism (24).

The aforementioned CHC is categorized as a non-selective MCT inhibitor, a group that also encompasses compounds such as phloretin, quercetin, p-CMBS, lonidamine, DIDs, and simvastatin (4). These non-selective MCT inhibitors are characterized by their limited specificity (61). Consequently, the development and utilization of selective MCT1 inhibitors assume heightened significance. AZD3965 stands out as one of the selective MCT1 inhibitors. Other selective MCT1 inhibitors include AR-C155858, BAY-8002, and 7ACC2 (24). Moreover, 3-bromopyruvate (3-BrPA) has been shown to induce an epigenetically driven loss of MCT1 (1). **Table 2** summarizes the efficacy of MCTs inhibitors.

**Table 2:** Efficacy of MCTs inhibitors.

Inhibitors	Inhibition index	MCT1	MCT2	MCT4	Reference
Non-selective MCT inhibitors					
Phloretin	Ki(um)	5	14	41	(4)
Quercetin	Ki(um)	10	5	40	(4)
p-CMBS	Ki(um)	25	NI	25	(4)
Lonidamine	Ki(um)	36	36	40	(4)
DIDs	Ki(um)	434	ND	NI	(4)
Simvastatin	Ki(um)	>200	ND	>200	(4)
CHC	Ki(um)	166	24	991	(4)
Selective MCT1 inhibitors					
AR-C155858	Ki(um)	0.002	<0.01	NI	(4)
AZD3965	Ki(um)	0.002	0.02	NI	(4)
BAY-8002	Ki(um)	Nanomolar range	Nanomolar range	NI	(4)
7ACC2	IC50(nm)	11 in SiHa cells	ND	ND	(39)
3-bromopyruvate	IC50(nm)	28.6 in HCT-116	/	/	(1)
		24.2 in UM-UC-3			
		14.7 in SiHa cells			

p-CMBS, p-chloromercuribenzenesulphonate; DIDs: 4,4′-diisothiocyanostilbene-2,2′-disulphonate; CHC, α-cyano-4-hydroxycinnamate; Ki, inhibition constant; IC50, half maximal inhibitory concentration; ND, not determined; NI, no inhibition;/, not mentioned.

**Figure 3:**
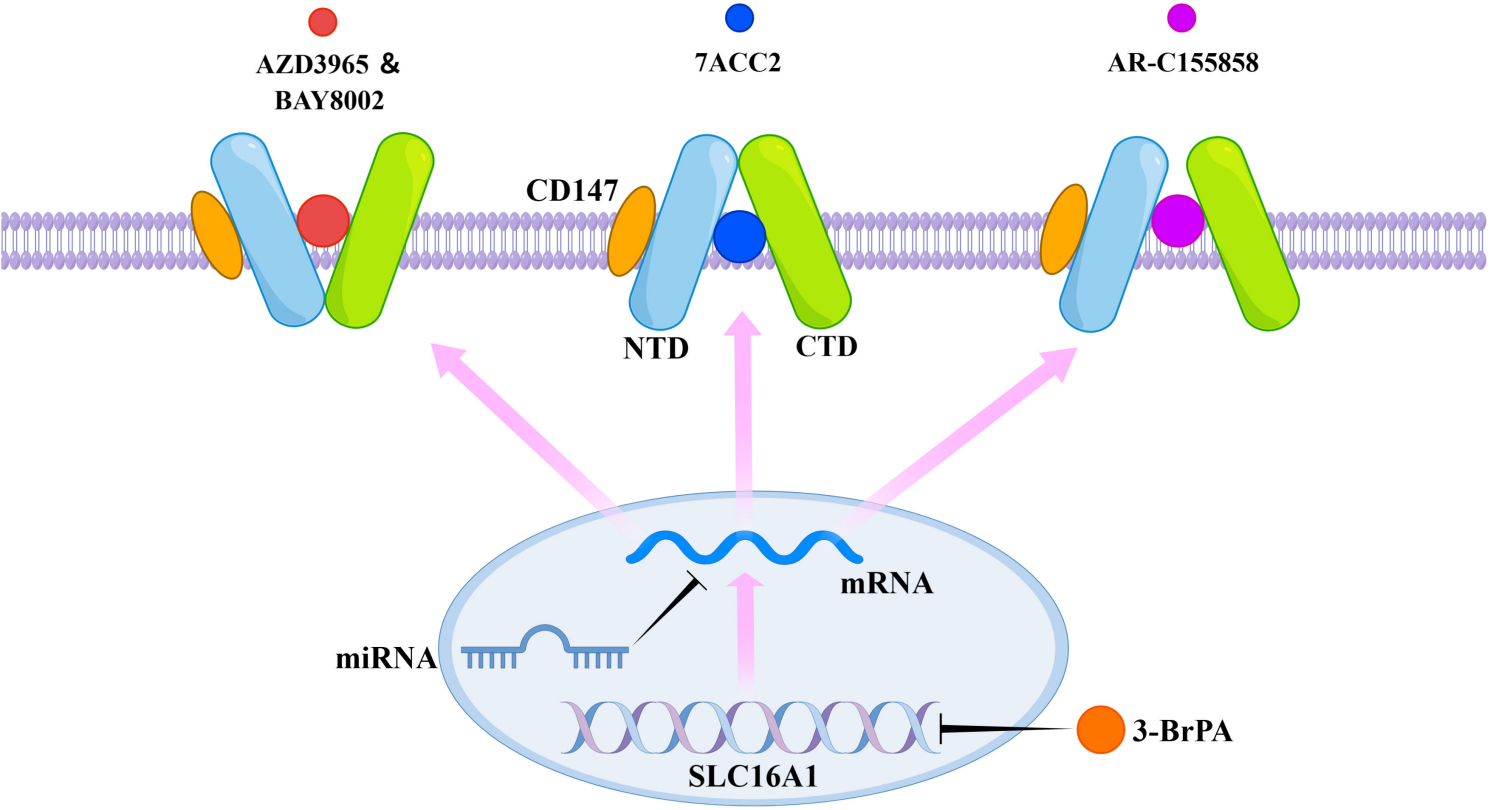
Mechanism of action of selective MCT1 inhibitors. AZD3965, BAY-8002, 7ACC2, and AR-C155858 exert their inhibitory effects by binding to specific sites of MCT1. 3-BrPA acts by inducing methylation of the gene coding for MCT1. MicroRNAs act by inhibiting the associated mRNAs.

AZD3965, BAY-8002, and 7ACC2 induce distinct conformational changes in MCT1, resulting in an outward-open conformation in the presence of BAY-8002 and AZD3965, and an inward-open conformation in the presence of 7ACC2 (61). All three inhibitors directly occupy the substrate binding site, achieving inhibition of MCT1 through direct competition with the substrate binding site and inhibition of conformational changes in the transporter protein (61). 3-BrPA treatment induces silencing of MCT1 by hypermethylating the promoter of the SLC16A1 gene (62). AR-C155858 interacts with transmembrane helices 7–10 in the inward-open conformation of MCT1, resulting in its inhibition (4). Additionally, microRNAs (miRNAs) such as miR-342-3p and miR-124 are found to downregulate MCT1 expression by targeting its messenger RNA (mRNA) (63, 64). MiR-146a indirectly inhibits the expression of MCT1 by inhibiting CD147 (65). The mechanism of action of MCT1 inhibitors is summarized in **Figure 3**.

## Relevant applications of selective MCT1 inhibitors

### AZD3965

AZD3965 is a pyrrole pyrimidine derivative (66). In a study involving B-cell lymphoma by Beloueche-Babari et al, treatment of immunodeficient mice with Raji xenografts using AZD3965 resulted in tumor growth inhibition and a reduction in choline levels (67). Mechanistically, intracellular lactate accumulation induced by AZD3965 led to decreased expression of choline kinase α (ChoKα) and its mRNA, consequently inhibiting *de novo* synthesis of choline phosphate (67). Simultaneously, there was an increase in the infiltration of dendritic cells (DCs) and natural killer cells (NKs) (67). In a systematic review assessing the anti-tumor properties of AZD3965 in a murine model, investigators determined that AZD3965 effectively augmented tumor responsiveness to radiation and chemotherapeutic agents (3).

### AR-C155858

AR-C155858 is also a pyrrole pyrimidine derivative (66). The use of AR-C155858 in combination with anti-CD19 chimeric antigen receptor (CAR)-T cell therapy has shown promising results in the treatment of B-cell malignancies (68). In a study focusing on B-cell lymphoma by Lopez et al, the inhibition of MCT1 by AR-C155858, in conjunction with CAR-T cells, led to enhanced *in vitro* cytotoxicity and improved anti-tumor control in a mouse model. Mechanistically, B-cell lymphoma cells primarily relied on MCT1 for lactate export and were susceptible to inhibition, with minimal to no expression of MCT4. Conversely, CAR-T cells exhibited elevated levels of both MCT1 and MCT4 following activation, rendering them functionally resistant to AR-C155858 inhibition (69). Furthermore, in the context of breast cancer by Guan et al, AR-C155858 has been shown to reduce tumor cell proliferation *in vitro* (70).

### 7ACC2

Malignant pleural effusion (MPE) is a common complication in advanced malignant tumors (71). FOXP3, a pivotal transcription factor, plays a crucial role in the regulation of immune responses. Within MPE, FOXP3 natural killer T (NKT)-like cells leveraged their elevated expression of MCT1 and LDHB to uptake and metabolize lactate, thereby maintaining their immunosuppressive capabilities within the effusion (72). *In vitro* studies have shown that 7ACC2 significantly reduced FOXP3 expression in NKT-like cells, which can inform the development of NKT-like cell-based therapies aimed at controlling MPE progression (72). In a study involving pancreatic cancer by Sandforth et al, tumor cells expressing MCT1 were protected against gemcitabine-induced apoptosis in a MCT1-dependent manner (73). The administration of 7ACC2 can counteract this protective effect and induce apoptosis in the tumor cells (73).

### BAY-8002

The DNA-dependent protein kinase catalytic subunit (DNA-PKcs) is known for its pleiotropic protein kinase activity and is often linked to poor prognosis in tumor patients (74). Wagner et al. revealed that overlocalization of DNA-PKcs can confer protection against lentiviral transduction, such as human immunodeficiency virus-1 (HIV-1). Meanwhile, BAY-8002, which inhibited lactate flux, enhanced the nuclear localization of DNA-PKcs, thereby reducing the efficacy of lentiviral transduction (75). In the context of prostate cancer by Matheux et al, MCT1 has been identified as a transporter for afatinib. The inhibition of MCT1 by BAY-8002 partially diminished the sensitivity of 22Rv1 cells, which expressed the pregnane X receptor, to afatinib (76).

## Challenges and coping strategies for MCT1 inhibitors

Although selective MCT1 inhibitors can effectively impede tumor progression, they also face notable challenges. For instance, in clinical trials, although AZD3965 was well tolerated, it was associated with side effects such as nausea and fatigue (1). In breast cancer research by Guan et al, AR-C155858 has been found to be ineffective in treating 4T1 xenograft breast tumor models, possibly due to alterations in the immune status of these preclinical models (70). Another study revealed that elevated levels of AR-C155858 can only impede cell proliferation without inducing cell death (77). Moreover, selective MCT1 inhibitors like AZD3965 and AR-C155858 have been shown to hinder lactate transport in tumor cells, but they did not significantly impede tumor progression (24).

Given these challenges, the integration of selective MCT1 inhibitors with other pharmaceutical agents may be a promising strategy in the realm of tumor therapy.

Selective MCT1 inhibitors have demonstrated promising therapeutic effects when used in combination with other drugs or treatment modalities. For instance, in a study involving hepatocellular carcinoma by Zhou et al, the combination of AR-C155858 with anti-PD-1 antibodies effectively suppressed tumor growth in xenograft models (78). Similarly, in the Raji Burkitt lymphoma model, combining AZD3965 with doxorubicin or rituximab resulted in decreased tumor growth (79). Furthermore, the combination of AZD3965 with various radiotherapy modalities has shown superior efficacy in treating tumor xenografts (80).

Another significant challenge faced by selective MCT1 inhibitors is their ineffectiveness in the presence of MCT4 overexpression (77). This may be due to reduced competition between MCT1 and MCT4 for CD147 (4). This limitation is particularly concerning because MCT4 is often co-expressed with MCT1 in tumors (81). Although AZD0095, a specific MCT4 inhibitor, has been developed (82), its efficacy has yet to be evaluated in clinical trials. Selective inhibitors targeting either MCT1 or MCT4 alone have proven ineffective against tumors that co-express both transporters, highlighting the need for further research into the development of dual MCT1 and MCT4 inhibitors for potential antitumor therapeutics (83). Syrosingopine, identified as a dual inhibitor of MCT1 and MCT4 (84), has demonstrated anti-proliferative effects in acute myeloid leukemia (85). Additionally, metformin, a drug commonly used to treat diabetes mellitus, has shown promise in the field of antitumor therapy (86). Metformin acts as an inhibitor of mitochondrial NADH dehydrogenase. NAD+ is essential for the glycolysis step that generates ATP, and it can be regenerated from NADH by either mitochondrial NADH dehydrogenase or lactate dehydrogenase. Syrosingopine increases intracellular lactate levels, which inhibits LDH activity. When metformin is combined with Syrosingopine, it blocks NAD+ regeneration, leading to glycolysis inhibition, ATP depletion, and ultimately, tumor cell death (77).

## Conclusive remarks

Overexpression of MCT1 in tumor cells is mostly associated with poor prognosis. MCT1 is thus implicated in promoting tumor progression via multiple mechanisms. Extensive research has elucidated that MCT1 and its mediated substrate transport not only sustain tumor progression through the maintenance of metabolic symbiosis but also promote angiogenesis, suppress the immune response of relevant immune cells, and engage in additional mechanisms that are conducive to tumor progression. A finding indicated that MCT1 expression in the cell membrane and MCT1 expression in the nucleus may predict divergent prognoses for patients. The nuclear localization of MCT1 may be associated with the binding of novel chaperone proteins. However, the precise mechanism underlying the nuclear localization of MCT1 remains to be elucidated and warrants further in-depth investigation. Moreover, it is also essential to explore whether other transport substrates of MCT1, beyond lactate and acetate, can promote tumor progression through specific mechanisms.

Given its pivotal role in tumor progression, MCT1 has garnered considerable attention as a potential therapeutic target for tumor treatment. Numerous studies have demonstrated that selective MCT1 inhibitors can inhibit tumor progression to a certain extent. However, these inhibitors also face several challenges. Therefore, further comprehensive studies are imperative to facilitate the development of MCT1-targeted therapeutic strategies.

## Glossary

3-BrPA: 3-Bromopyruvate
AMPK: adenosine monophosphate-activated protein kinase
APC: adenomatous polyposis coli
ATP: adenosine triphosphate
bFGF: basic fibroblast growth factor
CAR: chimeric antigen receptor
Cav-1: Caveolin-1
ccRCC: clear cell renal cell carcinoma
CHC: α-cyano-4-hydroxycinnamate
ChoKα: choline kinase α
CK1α: casein kinase 1 α
COX-2: cyclooxygenase-2
CRC: colorectal cancer
CTL: cytotoxic T lymphocyte
DC: dendritic cell
DLAT: dihydrolipoamide S-acetyltransferase
DNA-PKcs: DNA-dependent protein kinase catalytic subunit
DNMT: DNA methyltransferase
EE: endosome
EGFR-TKI: epidermal growth factor receptor tyrosine kinase inhibitor
ERK: extracellular regulated protein kinase
ETC: electron transport chain
FOXP3: forkhead box proteins P3
FZD: frizzled
G6P: fructose-6-phosphate
GLUT1: glucose transporter 1
GSK3: glycogen synthase kinase 3
HCAR1: hydroxy-carboxylic acid receptor 1
HIF-1: hypoxia-inducible factor 1
HIF-1α: hypoxia-inducible factor 1 α
HIV-1: human immunodeficiency virus-1
IκBα: inhibitor of κBα
IL-8: interleukin-8
LDH: lactate dehydrogenase
LKB1: liver kinase B1
LRP5/6: lipoprotein receptor‐related protein 5 and 6
MCT: monocarboxylate transporter
MCT1: monocarboxylate transporter 1
MCT4: monocarboxylate transporter 4
miRNA: microRNA
MMP: matrix metalloproteinase
MMR: mismatch repair
MPE: malignant pleural effusion
mRNA: messenger RNA
MUFA: monounsaturated fatty acid
MVB: multivesicular body
NAD: nicotinamide adenine dinucleotide
NADH: nicotinamide adenine dinucleotide
NFAT1: nuclear factor of activated T-cells 1
NF-κB: nuclear factor-kappa B
NK: natural killer cell
NKT: natural killer T
OSI: Osimertinib
OXPHOS: oxidative phosphorylation
PD-1: programmed death-1
PDC: pyruvate dehydrogenase complex
PDK1: pyruvate dehydrogenase kinase 1
PEP: phosphoenolpyruvate
PHD: prolylhydoxylase
SCD1: stearoyl-CoA desaturase 1
Smad3: recombinant mothers against decapentaplegic homolog 3
SREBP1: sterol regulatory element binding protein 1
STAT3: signal transducer and activator of transcription 3
TCF/LEF: T‐cell factor/lymphoid enhancer‐binding factor
TGF-β: transforming growth factor-β
TME: tumor microenvironment
Treg cell regulatory: T cell
VEGF-A: vascular endothelial growth factor A
VEGFR2: VEGF receptor 2.
